# Role of the Hypothalamic–Pituitary–Adrenal Axis in Health and Disease

**DOI:** 10.3390/ijms19040986

**Published:** 2018-03-26

**Authors:** Sharon DeMorrow

**Affiliations:** 1 Department of Medical Physiology, Texas A & M College of Medicine, Temple, TX 76504, USA; demorrow@medicine.tamhsc.edu; Tel.: +1-254-743-1299; 2 Central Texas Veterans Healthcare System, Temple, TX 76504, USA

The Hypothalamic–Pituitary–adrenal (HPA) axis describes a complex set of positive and negative feedback influences between the hypothalamus, pituitary gland, and adrenal gland. These positive and negative feedback mechanisms work in a neuroendocrine manner in order to modulate a number of physiological processes such as immunity, fertility, and the body’s response to stress. The mechanism by which the HPA axis remains in homeostasis depends widely on the release and uptake of several key regulatory molecules. The hypothalamus contains neuroendocrine neurons that secrete corticotropin-releasing hormone (CRH). CRH will, in turn, act on the pituitary gland to stimulate the production and release of adrenocorticotropic hormone (ACTH) into the circulation. Circulating ACTH then induces the adrenal gland to synthesize and release corticosteroids, such as cortisol and corticosterone. These circulating corticosteroids modulate the vast array of physiological processes influenced by the HPA axis and are also responsible for initiating a negative feedback loop on the HPA axis via activation of the glucocorticoid receptor (GR) in the brain in order to shut down corticosteroid production. This axis is dysregulated in a number of pathologies. The aim of this Special Issue was to provide an updated overview of how the HPA axis contributes to many different physiological and pathophysiological processes throughout the body. Experts in many fields have contributed to this far-reaching topic presented in this Special Issue “The role of the HPA axis in Heath and Disease”, published in the *International Journal of Molecular Sciences*. A total of 14 reviews and original articles have been published in this Special Issue delineating the various roles that the HPA axis plays in the physiology and pathology of different organs throughout the body and have been detailed in Table 1. I have categorized these manuscripts into the role of the HPA axis in (1) Pregnancy and early development; (2) neurobehavioral parameters, including anxiety and depression; (3) Other physiological responses; and (4) Pathophysiology of peripheral organs.

## 1. The Role of the HPA Axis in Pregnancy and Early Development

The Special Issue opens with the review article of Joseph and Whirledge [1] outlining the effects of the HPA axis on reproductive function and fertility. As part of the physiological adaptation to stress, the HPA axis mediates the function of the hypothalamic adrenal gonadal axis, which is responsible for the reproductive ability of an organism. The authors eloquently and concisely present the current understanding of the signaling mechanisms of the stress hormones of the HPA axis and their impact on fertility and introduce the current evidence of long-term effects of maternal stress during in utero development.

The topics covered in the first review have been nicely highlighted by the submission of two research articles. The first article, by Kalyani et al. [2] focused on the effects of pup separation on the stress response in postpartum female rats. The authors present data to suggest that there is a dampened HPA axis activity in response to stress in lactating rats when in the presence of their pups. However, after pup removal for 24 h, this stress response returned to pre-pregnancy state. The authors correlated the dampened stress response of the HPA axis in the presence of their pups in lactating rats to the high levels of circulating prolactin levels and increased prolactin receptor expression in the central nervous system and the restoration of the stress response to pre-pregnancy levels occurred once prolactin and prolactin receptor levels returned to their pre-pregnancy state. The second article by Schiffner et al. [3] hypothesizes that prenatal maternal exposure to low doses of the synthetic glucocorticoid dexamethasone may cause an alteration of the counter regulatory response of endogenous ACTH and cortisol during hypoglycemia. This hypothesis is based on the observations that stress hormones, such as ACTH and cortisol, stimulate gluconeogenesis and glucose release, that the activation of the HPA axis is related to metabolic syndrome and diabetes mellitus, and that hypoglycemia, with a resulting increase in ACTH and cortisol as a counter-regulatory response, is a common side effect of anti-diabetic treatment.

## 2. The Role of the HPA Axis in Neurobehavioral Parameters, Including Anxiety and Depression

The fourth article in this Special Issue, by Klimes-Dougan et al. [4], described the outcomes of a pilot project in which the function of the stress system was assessed in children with elevated socially withdrawn and/or aggressive behavior, and if the stress system function was predictively related to internalizing and externalizing problems for the high risk children. Compared to normally developing children, children with elevated socially withdrawn and/or aggressive behavior had a more blunted cortisol response to a stress paradigm consisting of a 5 min public speaking task and a 5 min mental arithmetic task. However, for the children in the high risk group, elevated cortisol levels at the start of the stress paradigm were associated with internalizing problems and predictive of improvement in internalizing problems over time. A complementary study in this Special Issue, by Chong et al. [5] was performed in emerging adults (defined as the period between adolescence and adulthood, or approximately 18 years old to mid-twenties), a period associated with a surge in the incidence of psychopathology, where the cortisol awakening response was assessed and compared to indexes of psychological functioning. Study limitations, implications and future directions of this study are discussed.

Next, in a research article by Lin et al. [6], the effects of Neuropeptide FF (NPFF) on the HPA axis and subsequent development of anxiety-like behaviors in rodents were assessed. Central NPFF, via activation of the NPFF receptor 2, increases HPA axis activity as assessed by serum corticosteroid levels. Furthermore, intracerebroventricular administration of NPFFR2 agonists increased c-Fos protein expression and induced anxiety-like behavior in these animals, indicating a direct and functional control of the HPA axis by NPFFR2 activation.

Lastly, in a correlative study, Doolin et al. [7] demonstrated that in patients suffering Major Depressive Disorder, there is a reduced morning cortisol and cortisol awakening response compared to healthy controls without any concomitant alteration in cortisone levels or the expression of cortisol/cortisone catalyzing enzyme 11β-hydroxysteroid dehydrogenase type I. Furthermore, there was a negative association between interleukin 1β mRNA in the blood and morning cortisol reactivity within the depressed group, indicating that dysregulation of the HPA axis and the immune system may be interconnected in patients with depression.

## 3. The Role of the HPA Axis in Other Physiological Responses

The sole manuscript in the section of this Special Issue devoted to the role of the HPA axis in physiological responses was a comprehensive review written by Azuma et al. [8] outlining the current knowledge of the association between the HPA axis and mastication. Specifically, studies have demonstrated that mastication influences hippocampal function by altering HPA axis activity and that chewing during stressful situations dampens the hyperactivity of the HPA axis. This review elegantly summarizes the molecular mechanisms involved in the interactions between mastication, hippocampal function and activity of the HPA axis.

## 4. The Role of the HPA Axis in the Pathophysiology of Peripheral Organs

This section of the Special Issue starts with a review by Cocco et al. [9] summarizing the incidence of specific autoantibodies against cells of the pituitary or hypothalamus in an array or diseases associated with the HPA axis. Our current knowledge and the limitations that preclude the use of specific autoantibodies of the hypothalamus or pituitary as diagnostic tool is eloquently discussed at length.

Next is a research article by Petrescu et al. [10] assessing the effects of glucocorticoid treatment of hepatic fibrosis in a rodent model of cholestatic liver injury. This work is based on the premise that HPA axis activity is suppressed in human and rodent models of liver disease and therefore treating cholestatic mice with low levels of glucocorticoids may prevent some of the liver pathology such as fibrosis. The authors also demonstrate that the anti-fibrotic effects of glucocorticoid treatment are sex-specific with a more pronounced therapeutic effect occurring in males.

The pathogenesis of pituitary adenomas, specifically prolactinomas are discussed in a review by Garcia-Barrado et al. [11]. Aromatase P450, the enzyme responsible for catabolizing aromatizable androgens to estrogens, is expressed throughout the body, including in the pituitary, is thought to be overexpressed in prolactinomas contributing to the pathogenesis of this tumor type. This review describes the synthesis of pituitary aromatase, its regulation of gonadal steroids and the physiological and pathophysiological roles of aromatase on the pituitary.

The cardiovascular system is another organ system particularly susceptible to prolonged dysregulation of the HPA axis. As is eloquently stated in the review by Burford et al. [12], questions exists as to whether cardiovascular health risks arise directly from the detrimental effects of prolonged stress axis activation, or are an indirect consequence of metabolic strain as a result of increased glucocorticoids. This review outlines the increasingly compelling evidence to suggest that a dysregulated HPA axis can play a direct role on the development of cardiovascular health risks.

The penultimate manuscript in this Special Issue is a review by Lin et al. [13], which discusses the role of the HPA axis in atopic dermatitis. This disorder is one of the most common chronic allergic inflammatory skin diseases which can be triggered or exacerbated by psychological stress. Furthermore, psychological stress has been shown to arise as a consequence of atopic dermatitis, thus setting up a vicious pathological cycle. Our current knowledge from both animal and human studies are thoroughly summarized and discussed in this review.

Lastly, from the array of articles and reviews outlined above, the use of synthetic glucocorticoids are a widely-prescribed therapy for the treatment of many diseases due to both the anti-inflammatory and immunosuppressive activities. However, if used in high doses for prolonged periods, systemic effects reminiscent of Cushing’s Syndrome can result. Conversely, after cessation of chronic prolonged glucocorticoid treatment, patients are at risk of developing tertiary adrenal insufficiency. A discussion of the potential symptoms, the pharmacological (e.g., type of compound, dose, duration of treatment etc.) and genetic factors that regulate the degree of HPA axis inhibition during synthetic glucocorticoid therapy are eloquently described in the comprehensive review by Paragliola et al. [14].

As the guest editor of this Special Issue with 14 contributions, I would like to thank all of the expert scientists who devoted their time and energy to preparing these manuscripts. These scientists were from a broad background of expertise, working in laboratories and research facilities throughout the world. For each submitted paper, the editorial team chose two or more experts in the field as external and independent reviewers whose recommendations were highly appreciated and substantially improved the quality of the articles in their final form. It was a pleasure for me to work with the authors and the competent editorial team to complete such an exciting project.

## Figures and Tables

**Table 1:** Summary of the papers in this Special Issue.

Authors	Title	Topic/Keywords	Type
Joseph and Whirledge [1]	Stress and the HPA Axis: Balancing Homeostasis and Fertility	Stress, fertility, reproduction, HPA axis, programming, glucocorticoids	Review
Kalyani et al. [2]	Effects of Pup Separation on Stress Response in Postpartum Female Rats	Lactation, prolactin, prolactin receptor, HPA axis, corticosterone, restraint stress	Article
Schiffner et al. [3]	Effects of Late Gestational Fetal Exposure to Dexamethasone Administration on the Postnatal Hypothalamus-Pituitary-Adrenal Axis Response to Hypoglycemia in Pigs	HPA axis, ACTH, cortisol, stress response, hypoglycemia	Article
Klimes-Dougan et al. [4]	A Pilot Study of Stress System Activation in Children Enrolled in a Targeted Prevention Program: Implications for Personalization	Children, prevention, intervention, cortisol, HPA axis, personalization	Article
Chong et al. [5]	Cortisol Awakening Response, Internalizing Symptoms, and Life Satisfaction in Emerging Adults	HPA axis, cortisol, cortisol awakening response, emerging adults, risk, life satisfaction	Brief report
Lin et al. [6]	NPFFR2 Activates the HPA Axis and Induces Anxiogenic Effects in Rodents	Neuropeptide FF, NPFF receptor 2, HPA axis, anxiety, paraventricular nuclei, corticotropin-releasing factor	Article
Doolin et al. [7]	Diurnal Hypothalamic-Pituitary-Adrenal Axis Measures and Inflammatory Marker Correlates in Major Depressive Disorder	Major depressive disorder, hypothalamic-pituitary-adrenal axis, immune system, cortisol awakening response, inflammation,	Article
Azuma et al. [8]	Association between Mastication, the Hippocampus, and the HPA Axis: A Comprehensive Review	Chewing, masticatory dysfunction, hippocampus, HPA axis	Review
Cocco et al. [9]	The Hypothalamic–Pituitary Axis and Autoantibody Related Disorders	Autoimmunity, pituitary, hypothalamus, autoantibodies	Review
Petrescu et al. [10]	Glucocorticoids Cause Gender-Dependent Reversal of Hepatic Fibrosis in the MDR2-Knockout Mouse Model	HPA axis, corticosterone, glucocorticoid receptor, hepatic cholestasis, liver fibrosis, cholangiocytes	Article
Garcia-Barrado et al. [11]	Relation among Aromatase P450 and Tumoral Growth in Human Prolactinomas	Pituitary gland, aromatase, prolactinoma	Review
Burford et al. [12]	Hypothalamic-Pituitary-Adrenal Axis Modulation of Glucocorticoids in the Cardiovascular System	HPA axis, glucocorticoids, glucocorticoid receptor, mineralocorticoid receptor, smooth muscle cells, vascular endothelial cells, cardiomyocytes, heart	Review
Lin et al. [13]	Association between Stress and the HPA Axis in the Atopic Dermatitis	Atopic dermatitis, HPA axis, psychological stress, skin barrier, inflammation, glucocorticoid	Review
Paragliola et al. [14]	Treatment with Synthetic Glucocorticoids and the Hypothalamus–Pituitary–Adrenal Axis	Synthetic glucocorticoid, tertiary hypoadrenalism, iatrogenic Cushing’s syndrome	Review

## Abbreviations

ACTH	Adrenocorticotropic hormone
CRH	Corticotropin releasing hormone
GR	Glucocorticoid receptor
HPA	Hypothalamic pituitary adrenal axis
NPFF	Neuropeptide FF
